# Secreted NS1 of Dengue Virus Attaches to the Surface of Cells via Interactions with Heparan Sulfate and Chondroitin Sulfate E

**DOI:** 10.1371/journal.ppat.0030183

**Published:** 2007-11-30

**Authors:** Panisadee Avirutnan, Lijuan Zhang, Nuntaya Punyadee, Ananya Manuyakorn, Chunya Puttikhunt, Watchara Kasinrerk, Prida Malasit, John P Atkinson, Michael S Diamond

**Affiliations:** 1 Department of Medicine, Washington University School of Medicine, St. Louis, Missouri, United States of America; 2 Medical Molecular Biology Unit, Office for Research and Development, Mahidol University, Bangkok, Thailand; 3 Department of Pathology and Immunology, Washington University School of Medicine, St. Louis, Missouri, United States of America; 4 Department of Pathology, Faculty of Medicine Siriraj Hospital, Mahidol University, Bangkok, Thailand; 5 Medical Biotechnology Unit, National Center for Genetic Engineering and Biotechnology BIOTEC, National Science and Technology Development Agency NSTDA, Pathumthani, Thailand; 6 Department of Medical Technology, Faculty of Associated Medical Sciences, Chiang Mai University, Chiang Mai, Thailand; 7 Department of Molecular Microbiology, Washington University School of Medicine, St. Louis, Missouri, United States of America; Scripps Research Institute, United States of America

## Abstract

Dengue virus (DENV) nonstructural protein-1 (NS1) is a secreted glycoprotein that is absent from viral particles but accumulates in the supernatant and on the plasma membrane of cells during infection. Immune recognition of cell surface NS1 on endothelial cells has been hypothesized as a mechanism for the vascular leakage that occurs during severe DENV infection. However, it has remained unclear how NS1 becomes associated with the plasma membrane, as it contains no membrane-spanning sequence motif. Using flow cytometric and ELISA-based binding assays and mutant cell lines lacking selective glycosaminoglycans, we show that soluble NS1 binds back to the surface of uninfected cells primarily via interactions with heparan sulfate and chondroitin sulfate E. DENV NS1 binds directly to the surface of many types of epithelial and mesenchymal cells yet attaches poorly to most peripheral blood cells. Moreover, DENV NS1 preferentially binds to cultured human microvascular compared to aortic or umbilical cord vein endothelial cells. This binding specificity was confirmed in situ as DENV NS1 bound to lung and liver but not intestine or brain endothelium of mouse tissues. Differential binding of soluble NS1 by tissue endothelium and subsequent recognition by anti-NS1 antibodies could contribute to the selective vascular leakage syndrome that occurs during severe secondary DENV infection.

## Author Summary

Dengue virus (DENV) is a mosquito-transmitted virus that infects humans and has become a global emerging infectious disease threat. Four serotypes of DENV exist, and the most severe cases are associated with secondary infection with a different virus serotype. Clinical deterioration is characterized by bleeding and selective vascular leakage from endothelium in specific tissue sites. An increased understanding of how DENV proteins contribute to this phenotype is vital to developing novel vaccines and identifying individuals at risk for severe disease. DENV nonstructural protein-1 (NS1) is one such protein: during infection, it is secreted and accumulates in the supernatant and on the surface of cells. In this study, we demonstrate that soluble DENV NS1 attaches to subsets of cells, including some but not all endothelial cells, primarily via an interaction with specific glycosaminoglycans (heparan sulfate and chondroitin sulfate E). This was confirmed in tissue binding studies as DENV NS1 bound to lung and liver but not intestine or brain endothelium. Our findings suggest that the selective vascular leakage that occurs in severe DENV infection may be related to the relative ability of endothelial cells in different tissues to bind soluble NS1 and to be targeted by cross-reactive anti-NS1 antibodies during secondary infection.

## Introduction

Dengue hemorrhagic fever and dengue shock syndrome (DHF/DSS) are severe and potentially fatal complications of infection by dengue virus (DENV), a mosquito-borne RNA virus of the Flaviviridae family. Globally, DENV infects 25 to 100 million people per year, but the life-threatening complications primarily occur in school-age children [1]. Four serotypes of DENV exist, and DHF/DSS is commonly associated with secondary infection with a different virus serotype [2,3]. In the most severe cases, clinical deterioration is characterized by a rapid decline after several days of continuous high fever, thrombocytopenia, and selective vascular leakage at serosal sites [4]. The vascular leakage syndrome results in hemoconcentration, pleural effusions and ascites, and hypotension [4]. An effective strategy for disease prevention or treatment is currently lacking.

The pathogenesis of DHF/DSS reflects a complex interplay of the host immune response and the viral determinants of virulence [5–7]. A model of immunopathogenesis has been suggested based on an increased risk of DHF with secondary infection and in children within the first year of life born to DENV-immune mothers [8,9]. From this, the hypothesis of antibody-dependent immune enhancement of infection emerged. In support of it, enhancement of DENV infection in monocytes in vitro with pre-illness serum correlates with increased risk of DHF in vivo [9,10], and peak viremia is higher in patients with severe secondary DENV infection [11–13]. Differences in specific genetic determinants among viral isolates [14–16] also modulate virulence, as some DENV strains fail to cause severe disease [17,18]. A pathologic cytokine response that occurs after extensive T cell activation also likely contributes to the syndrome [5]. Elevated levels of cytokines including IFN-α, TNF- α, and IL-10 correlate with severe disease [19–24], and the expansion of cross-reactive low-affinity DENV-specific T cells produces vasoactive cytokines [25–27]. Finally, accumulation of soluble NS1 in serum also correlates with disease severity and is believed to contribute to changes in vascular permeability through antibody-dependent activation of the complement cascade [28].

NS1 is a ∼48-kDa glycoprotein that is absent from the infectious viral particle. NS1 is, however, an essential gene within infected cells, as it functions as a cofactor for viral RNA replication, colocalizing with the double-stranded RNA replicative form [29,30]. NS1 is synthesized in an infected cell as a soluble monomer and rapidly dimerizes after post-translational modification in the lumen of the endoplasmic reticulum, with subsequent transport to the cell surface and release into extracellular milieu [31]. In solution, secreted NS1 behaves as a hexamer [32] and accumulates in serum in high amounts (up to 50 μg/ml) [11,28,33]. The mechanism(s) by which soluble and cell surface–associated NS1 contributes to flavivirus pathogenesis remains uncertain. It has been proposed to facilitate immune complex formation [28,34], elicit auto-antibodies that react with platelet and extracellular matrix proteins [35,36], cause endothelial cell damage via antibody-dependent complement-mediated cytolysis [35,37–39], directly enhance infection [40], and attenuate the alternative pathway of complement activation by binding factor H [41].

The mechanism by which NS1 becomes associated with the plasma membrane is poorly understood. The protein lacks amino- or carboxyl-terminal membrane-spanning or anchoring sequences [42]. Although covalent linkage by a glycosylphosphatidylinositol (GPI) anchor to the plasma membrane of DENV-infected cells has been suggested [43], this mechanism has not been confirmed. Also, it does not explain how uninfected, as well as infected cells, accumulate NS1 on their surfaces. In this study, we demonstrate that soluble DENV NS1 attaches to uninfected cells primarily via an interaction with the glycosaminoglycans (GAG) heparan sulfate (HS) and chondroitin sulfate E (CS-E), and that GAG sulfation is a critical cellular modification necessary for binding. Moreover, soluble DENV NS1 preferentially binds subsets of cells including human microvascular endothelial cells. Our findings suggest that the selective vascular leakage that occurs in severe DENV infection may be related to the relative ability of endothelial cells in different tissues to bind soluble NS1 and be targeted by cross-reactive anti-NS1 antibodies during secondary infection.

## Results

**Figure 1:**
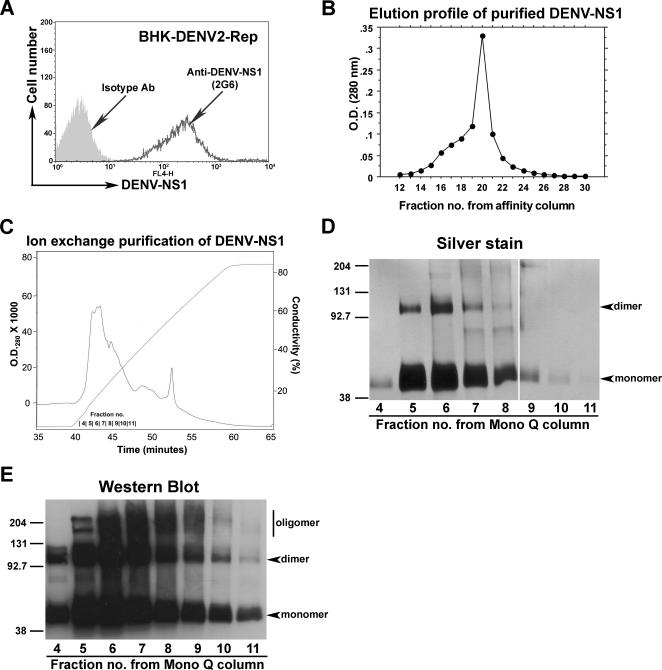
Purification of DENV NS1 from BHK DENV-2 Rep Cells (A) A flow cytometry histogram of expression of DENV NS1 in BHK DENV-2 Rep cells. Cells were fixed and stained with anti-DENV-2 NS1 mAbs or isotype control Abs. One representative experiment of three is shown. (B) Elution profile of purified NS1 after immunoaffinity chromatography of serum-free supernatants of BHK DENV-2 Rep cells. Peak fractions (19–21) were combined and passed over an ion-exchange column, and purified NS1 was eluted (C) with a linear NaCl gradient (diagonal line). (D) SDS-PAGE of purified soluble DENV NS1 from BHK DENV-2 Rep cells. Purified NS1 was boiled prior to nonreducing 12% SDS-PAGE and silver staining. Molecular weight markers are shown at the left. (E) Immunoreactivity of purified DENV-2 NS1. Western blot analysis of purified DENV-2 NS1 after nonreducing 12% SDS-PAGE was performed using the 2G6 anti-DENV NS1 mAb.

Flavivirus NS1 is a secreted nonstructural glycoprotein that lacks a membrane-spanning region yet becomes cell surface–associated [31,42]. As NS1 attachment to the cell surface may be important for immune recognition [28,44] and immune evasion [41], we set out to identify the mechanism by which soluble NS1 binds to the surface of cells. Although it has been suggested that DENV NS1 attaches to the plasma membrane via a GPI anchor [43], this is unlikely to explain how DENV NS1 binds to uninfected cells. To assess this issue in greater detail, we developed a flow cytometric–based NS1 cell surface binding assay. We purified soluble DENV NS1 from the supernatants of baby hamster kidney (BHK) cells that stably express a DENV-2 subgenomic replicon (BHK DENV-2 Rep cells [45]). These cells produce high levels of intracellular (Figure 1A) and soluble NS1 as determined by a capture ELISA (3.6 ± 0.2 μg/ml of supernatant) and western blot (unpublished data). Elution profiles of purified NS1 after immunoaffinity or ion exchange chromatography are shown (Figure 1B and 1C, respectively). The yield of purified NS1 from BHK DENV-2 Rep cells (Figure 1D) was similar to that previously obtained from DENV-2-infected cell supernatants [28] and retained immunoreactivity as judged by western blot (Figures 1E and S1) or ELISA with a panel of conformationally sensitive anti-NS1 mAbs (unpublished data).

### DENV NS1 Selectively Binds to Uninfected Cells of Specific Lineage

**Figure 2:**
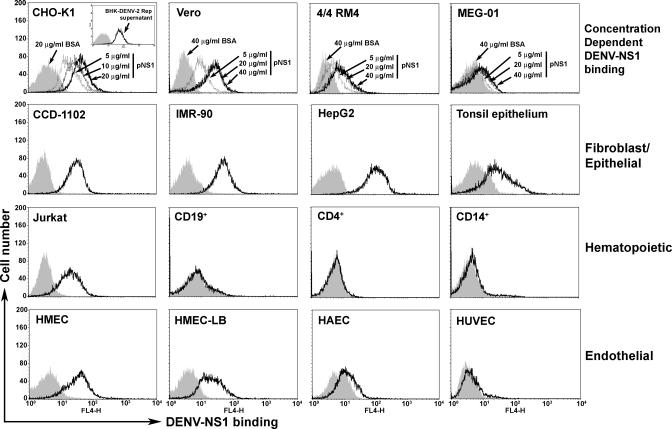
DENV NS1 Binds to Cells of Specific Lineages Dose-dependent binding of DENV NS1 to cell surfaces. Purified DENV NS1 (5, 10, 20, and 40 μg/ml) was added to different cell types at 4 °C for 1 h. After washing, bound NS1 was detected by anti-NS1 antibody staining and flow cytometry. An example of DENV NS1 binding to CHO-K1 cells using supernatant from BHK-DENV-2 Rep cells is depicted in the inset at the upper right corner of the top left histogram. Examples of histogram profiles of DENV NS1 binding to epithelial, fibroblast, hematopoietic, and endothelial cells are depicted. One representative of three experiments is shown. No specific staining was observed after BSA binding and incubation with an NS1 antibody or NS1 binding and incubation with an isotype-matched control antibody (unpublished data).

Uninfected cells were tested for the ability to bind soluble DENV NS1. Incubation with either purified DENV NS1 or supernatants from BHK DENV-2 Rep cells resulted in rapid binding of NS1 to many cell types (Figure 2 and Table 1). Binding of NS1 to Chinese hamster ovarian epithelial (CHO)-K1, Vero, and 4/4 RM4 cells was dose-dependent and saturable, and maximum binding was achieved at a concentration of 20 μg/ml, which is in the range reported in vivo during secondary infection [11,28]. DENV NS1 bound to the surface of several types of epithelial and fibroblast transformed cell lines (BHK, CHO-K1, Vero, 293T, HepG2, Hep3B, and L929) including those of human and nonhuman origin. NS1 also bound to primary, untransformed cells including keratinocytes (HaCat, CCD-1102), skin and lung fibroblasts (Detroit-551 and IMR-90), and freshly isolated tonsillar epithelial cells.

**Table 1:**
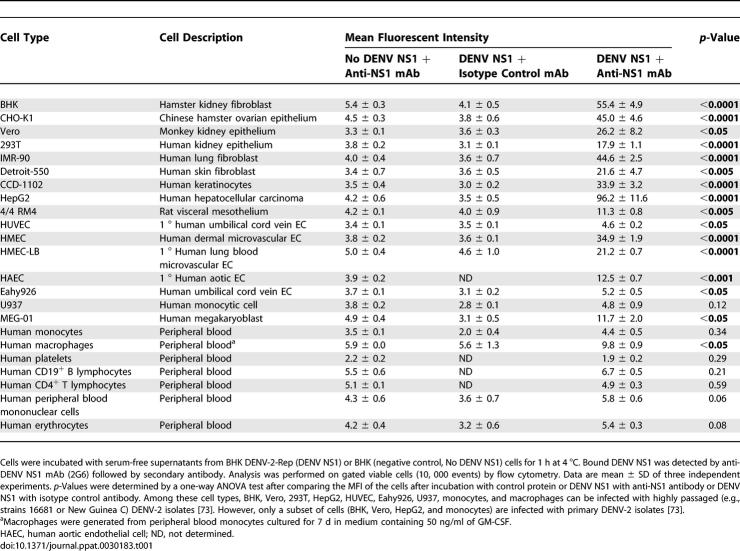
DENV NS1 Binds to a Variety of Cell Types

As high levels of soluble NS1 are detected in the blood of DHF patients [11,28,33], we extended our analysis to human peripheral blood cells. Notably, DENV NS1 failed to bind to the surface of freshly isolated peripheral blood mononuclear cells or erythrocytes. Similar negative results were obtained with purified CD14^+^ monocytes, CD19^+^ B lymphocytes, and CD4^+^ T lymphocytes. Moreover, DENV NS1 only weakly bound to the surface of monocyte-derived macrophages. In contrast to that observed with primary lymphocytes, DENV NS1 bound strongly to the surface of several malignant T cell lines, including Jurkat, H9, and EL-4 (Figure 2 and unpublished data).

As vascular leakage is a hallmark of DHF/DSS, and endothelial cells are believed to be targets of immune-mediated damage, we analyzed the binding of NS1 to human endothelial cells. Interestingly, DENV NS1 bound strongly to human dermal and lung microvascular endothelial cells (HMEC) and HMEC-lung blood (HEMC-LB), modestly to aortic endothelial cells, but minimally to primary or immortalized human umbilical vein endothelial cells (HUVEC or Eahy926).

### Soluble DENV NS1 Binds to GAG

**Figure 3:**
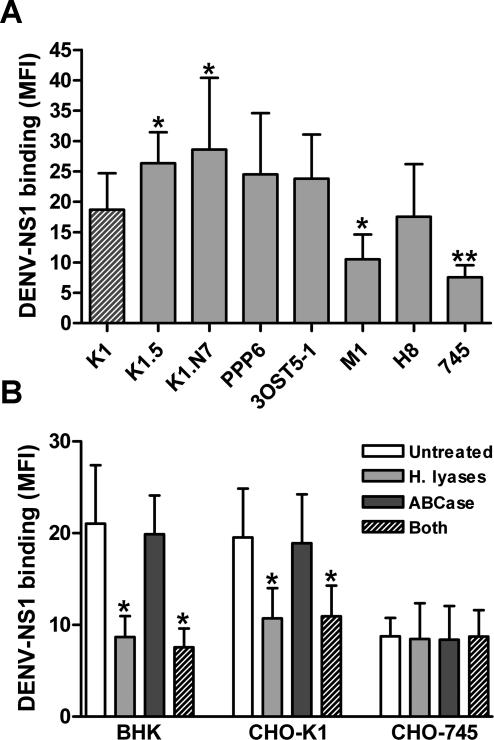
Binding of DENV NS1 to Cell Surface Is GAG-Dependent (A) DENV NS1 binding to wild type (K1) and seven mutant CHO cell lines that overexpress (K1.5, K1.N7, PPP6, and 3OST5–1) or are defective (M1, H8, and 745) for specific enzymes in GAG synthesis. Binding and staining were performed at 4 °C and analyzed by flow cytometry. Data are expressed as the specific MFI of staining after subtraction of background antibody binding in the absence of NS1. The error bars indicate standard deviation (SD) corresponding to three independent experiments. Asterisks note NS1 binding that is statistically different from the CHO-K1 wild-type cells (* *p* < 0.05, ** *p* < 0.005). (B) Cell surface HS and CS were enzymatically removed from BHK or wild-type CHO-K1 cells after treatment with a mixture of heparin lyases I, II, III (H. lyases) or chondroitinase ABC (ABCase) and assayed for binding of DENV NS1. A GAG-negative mutant cell (CHO-745) was used as a negative control. Analysis was performed by flow cytometry. Data are expressed as the specific MFI of staining after subtraction of background antibody binding in the absence of NS1. The error bars indicate SD corresponding to three independent experiments. Asterisks indicate DENV-2 NS1 binding that is statistically different from untreated cells (* *p* < 0.05, ** *p* < 0.005).

As our data indicated that soluble DENV NS1 bound a subset of uninfected mammalian cells of different lineages, we sought to identify the mechanism of attachment. We hypothesized that NS1 might interact with a highly conserved moiety, such as a GAG. To test this, we compared DENV NS1 binding to wild-type CHO-K1 cells and seven different CHO cell lines (Table 2) that are either defective in GAG biosynthesis or express GAG with distinct structural specificities. CHO-745 cells, which genetically lack xylosyltransferase, the enzyme required for biosynthesis of both HS and CS [46], showed a 60%–70% reduction (*p* < 0.0001) in binding of DENV NS1 (Figure 3A and Table 2). Similarly, CHO-M1 cells, which are defective in HS biosynthesis [47], exhibited a ∼50% reduction (*p* = 0.02) in DENV NS1 binding. However, CHO-H8 cells [48], which are a variant of CHO-M1 HS-deficient cells overexpressing CS 2-*O*-sulfotransferase (2-OST) (producing a 30-fold increase in double sulfated disaccharide residues on CS), showed levels of DENV NS1 binding that were similar to CHO-K1 wild-type cells. Thus, both HS and CS sustain cell surface binding to DENV NS1, at least on CHO cells.

**Table 2:**
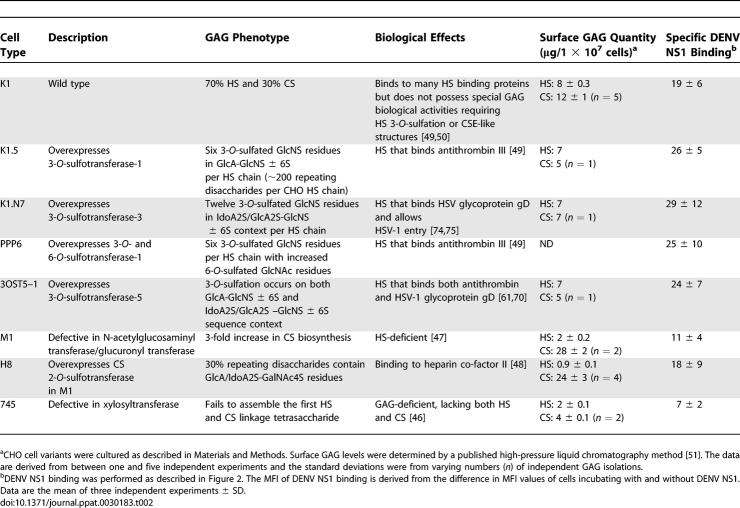
Description of Wild Type and Mutant CHO Cells

To determine if DENV NS1 required modification of HS structures for binding, we tested CHO cell lines that overexpress different 3-*O*-sulfotransferases (3-OST) (Figure 3A and Table 2). All 3-OSTs transfer sulfate moieties to the 3-*O*-position of N-sulfated glucosamine (GlcNS) and generate a highly sulfated HS motif with distinct biological properties (Table 2). CHO-K1.5 cells overexpress 3-OST-1, which adds 3-*O*-sulfate to GlcNS in a glucuronic acid (GlcA)-GlcNS ± 6S sequence context, and facilitate high affinity antithrombin III binding [49]. CHO-K1.N7 cells overexpress 3-OST-3, which adds 3-*O*-sulfate to GlcNS in a GlcA2S/iduronic acid (IdoA) 2S-GlcNS ± 6S sequence context, and promote herpes simplex virus (HSV) glycoprotein D binding and entry [50]. By contrast, overexpression of 3-OST-5 (CHO-3-OST-5 cells) adds 3-*O*-sulfate to GlcNS in both GlcA-GlcNS ± 6S and GlcA2S/IdoA2S-GlcNS ± 6S sequence contexts. Notably, all CHO cell lines expressing distinct 3-OST showed enhanced DENV NS1 binding by 30%–50% (*p* < 0.05) without a preference for any of the distinct 3-OST-modified HS structures. Thus, unlike other glycoproteins, DENV NS1 prefers highly sulfated HS motifs for binding and does not require specific HS sequences.

On CHO cell lines, the specificity of the GAG had a more dominant effect on regulating NS1 binding than the absolute level. Using a previously developed high-pressure liquid chromatography method [51], we quantified the amount of cell surface HS and CS GAG in different CHO cell lines and compared it to NS1 binding (Table 2). Although CHO-M1 cells expressed twice the amount of total GAG compared to wild-type CHO-K1 cells, CHO-M1 cells bound NS1 poorly. Thus, the total level of GAG did not directly correlate with NS1 binding. Consistent with this, similar amount of GAG were observed on CHO-K1 and other 3-OST-expressing CHO cell lines, yet 3-OST-expressing CHO cells bound NS1 more strongly. Moreover, Vero cells express ∼3-fold higher levels of HS compared to CHO-K1 cells (unpublished data) but bound NS1 less well compared to CHO-K1 cells (Figure 2 and Table 2).

A requirement of GAG for DENV NS1 binding to the cell surface was confirmed independently by enzymatic treatment of BHK and CHO-K1 cells with specific heparin lyases and chondroitinases (Figure 3B). Treatment of BHK or CHO-K1 cells with heparin lyases I, II, and III, which specifically remove HS, reduced DENV NS1 binding (∼50%–60%, *p* < 0.05) to the levels observed in GAG-negative CHO-745 cells. In contrast, DENV NS1 attachment was not significantly affected by treatment with chondroitinase ABC, which degrades all types of CS [52]. As expected, treatment with both enzymes had no effect on DENV NS1 binding to CHO-745 cells, which lack GAG. Nonetheless, a small amount of NS1 binding to CHO-745 cells was observed, presumably through a subordinate GAG-independent pathway. Overall, the analysis of CHO cell mutants and enzymatic treatments suggested that HS and highly sulfated forms of CS were the primary cell surface GAG that sustained DENV NS1 binding on CHO and BHK cells.

**Figure 4:**
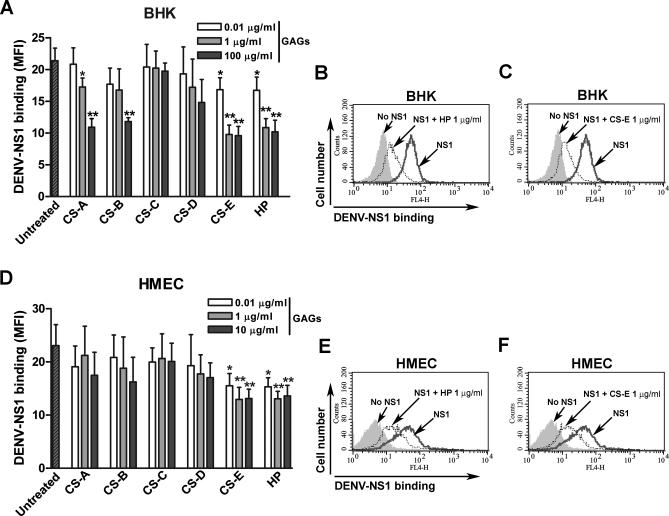
Soluble HP and CS-E Block DENV NS1 Binding to the Surface of BHK and HMEC DENV NS1 was incubated with three concentrations of indicated soluble GAG prior to incubation with (A–C) BHK or (D–F) HMEC. DENV NS1 was detected by flow cytometry (as described in Figure 2). Data are expressed as the specific MFI of staining as described in Figure 3. The error bars indicate SD corresponding to three independent experiments. Asterisks denote NS1 binding that is statistically different from untreated cells (* *p* < 0.05, ** *p* < 0.005). Examples of histogram profiles in which soluble HP and CS-E at 1 μg/ml concentration block DENV NS1 binding to the surface of (B–C) BHK and (E–F) HMEC are depicted.

**Figure 5:**
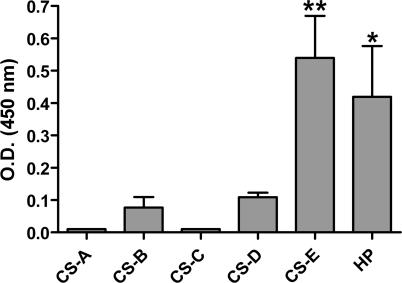
Direct Binding of DENV NS1 with HS and CS-E Microtiter plates were coated with indicated soluble GAG overnight at 4 °C. After incubation with supernatants from BHK-DENV-2-Rep cells or control BHK cells, bound DENV NS1 was detected using DENV NS1–specific mAbs, followed by biotin-conjugated anti-mouse IgG and HRP-conjugated streptavidin. Plates were read at an optical density of 450 nm on a 96-well plate reader. Data are the mean ± SD from four independent experiments. Asterisks denote NS1 binding that is statistically different from binding to background (* *p* < 0.05, ** *p* < 0.005). O. D., optical density.

As an additional confirmation, we performed competitive binding assays with soluble GAG. Pre-incubation with soluble CS-E or heparin (HP) substantially decreased (∼50%, *p* < 0.005), in a dose-dependent manner, DENV NS1 binding to BHK cells (Figure 4A–4C). Small, albeit significant inhibition (∼15%, *p* < 0.05), was observed even at relatively low concentrations CS-E and HP (0.01 μg/ml or 0.033 nM). In contrast, CS-A and -B inhibited NS1 binding only at higher concentrations (100 μg/ml or 3.3 μM, *p* < 0.05) whereas CS-C and -D showed no significant effect (*p* > 0.2). Similarly, pre-incubation with CS-E and HP reduced DENV NS1 attachment to HMEC cells (∼40%, 0.01 μg/ml, *p* < 0.01) (Figure 4D–4F). In a more direct in vitro binding ELISA, an interaction between DENV NS1 and HS or CS-E was established, whereas only weak or no appreciable binding was detected with CS-A, -B, -C, and -D (Figure 5). Taken together, these results suggest that NS1 interacts more strongly with HP, HS, and CS-E and more weakly with CS-A and CS-B. CS-A consists of mainly GlcA-GalNAc4S repeating disaccharides, CS-B contains both IdoA-GalNAc4S and GlcA-GalNAc4S as dominant repeating disaccharides, and CS-C consists of mainly GlcA-GalNAc6S repeating disaccharides. CS-D and CS-E are enriched in disulfated disaccharides, GlcA2S-GalNAc6S and GlcA-GalNAc4S6S, respectively. CS-E is unique, as two sulfates are present in the same GalNAc residue compared to other CS, and suggests that clustered sulfates rather than net negative charge on the disaccharide backbone facilitate DENV NS1 binding.

### GAG Sulfation Is Critical for DENV NS1 Cellular Attachment

**Figure 6:**
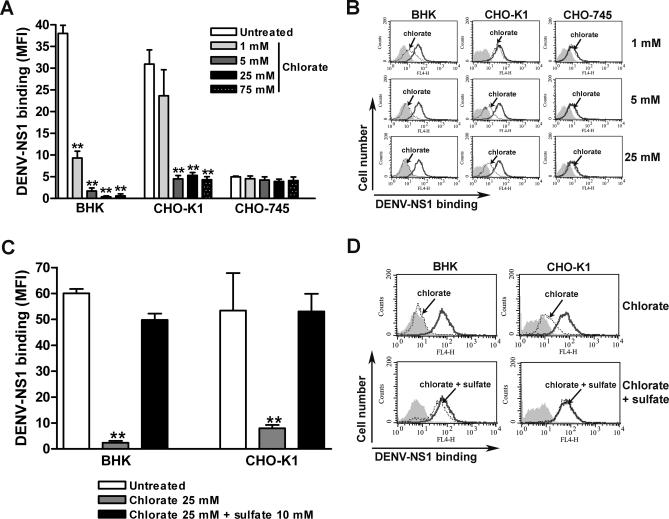
Sulfation Is Required for Soluble DENV NS1 Binding to Cells (A–B) BHK, wild-type CHO-K1, and GAG-deficient mutant CHO-745 were cultured overnight in sulfate-free medium in the presence of indicated amounts of sodium chlorate. Cells were harvested and tested for NS1 binding by flow cytometry as described in Figure 2. No appreciable cell death was observed by this treatment as judged by exclusion of propidium iodide. Data (A) are expressed as the specific MFI of staining as described in Figure 3. The error bars indicate SD corresponding to three independent experiments. Asterisks denote NS1 binding that is statistically different from untreated cells (** *p* < 0.005) (C–D) Addition of sodium sulfate (10 mM) restores binding of NS1 to cells. Cells were cultured overnight in sulfate-free medium and 25 mM sodium chlorate with or without 10 mM sodium sulfate (as indicated). Cells were processed for DENV NS1 binding and analyzed by flow cytometry (see Figure 2). Data (C) are expressed as the specific MFI of staining as described in Figure 3. The error bars indicate SD corresponding to three independent experiments. Asterisks denote NS1 binding that is statistically different from untreated cells (** *p* < 0.005).

The specificity of GAG-protein interactions often depends on the position and degree of sulfation (reviewed in [53–55]). To clarify if GAG sulfation is required for cell binding of DENV NS1, we treated BHK and CHO-K1 cells with the reversible sulfation inhibitor, sodium chlorate [56] (Figure 6A and 6B). This treatment did not alter cell viability, as analyzed by propidium iodide exclusion (unpublished data). However, binding of DENV NS1 to BHK cells was abolished by the addition of sodium chlorate (>5 mM). In contrast, at comparable doses, DENV NS1 binding to wild-type CHO-K1 was reduced to the level observed with CHO-745 cells lacking GAG. Importantly, the phenotype was reversed by the addition of excess exogenous (10 mM) sodium sulfate (Figure 6C and 6D). Thus, GAG sulfation is required for optimal binding of soluble DENV NS1 to cells.

### Expression of DENV NS1 on the Surface of DENV-Infected Cells Is Largely GAG-Independent

**Figure 7:**
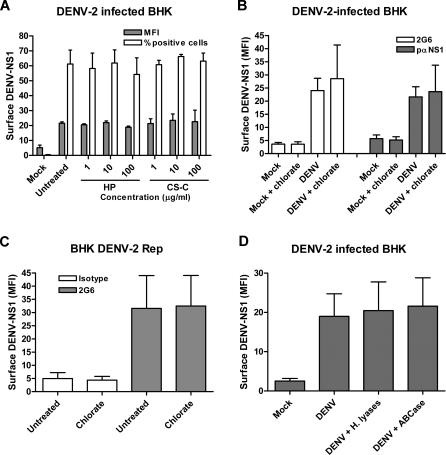
Expression of DENV NS1 on the Surface of Infected Cells Is Not Dependent on GAG (A) BHK cells were infected with DENV-2 at the MOI of 3 and cultured for 24 h in the presence of indicated concentrations of soluble HP or CS-C. Cells were then harvested for analysis of surface NS1 expression. (B) BHK cells were infected with DENV-2 at the MOI of 3 and cultured for 24 h in the presence of 25 mM sodium chlorate. DENV NS1 expression was determined and analyzed by flow cytometry using anti-DENV NS1 mAb (2G6) or polyclonal anti-DENV NS1. (C) BHK DENV-2 Rep cells were cultured in medium containing 25 mM sodium chlorate for 48 h. Expression of NS1 on the cell surface was determined by using anti-DENV NS1 mAb (2G6) and analyzed by flow cytometry. (D) BHK cells were infected with DENV-2 at the MOI of 3. At 24 h after infection, the cells were treated with a mixture of heparin lyases I, II, III (H. lyases) or chondroitinase ABC (ABCase). NS1 expression was analyzed by flow cytometry using the 2G6 anti-DENV NS1 mAb. Data are the mean ± SD of three independent experiments as described in Figure 6.

Although our data suggests that soluble DENV NS1 binds to the cell surface of uninfected cells via HS and CS-E, we evaluated if this was the dominant mechanism of attachment of NS1 to DENV-infected cells. Previous studies have suggested that flavivirus NS1 may be displayed on the cell surface of infected cells by a GPI anchor or transmembrane linkage [43], even though the protein lacks anchor attachment sequences or consensus membrane spanning domains. To evaluate this, BHK cells were infected with DENV-2 and cultured in the presence of high concentrations of soluble HP. No reduction of DENV NS1 surface expression on infected cells was observed (Figure 7A). These results were confirmed by studies with the sulfate inhibitor, sodium chlorate. In contrast to the inhibitory effect of sodium chlorate on soluble NS1 binding (Figure 6), no reduction of DENV NS1 expression on the surface of infected BHK cells was observed (Figure 7B). Similar studies with BHK DENV-2 Rep cells also showed no change in surface NS1 expression after sodium chlorate treatment (Figure 7C). Finally, in direct contrast to that observed with soluble NS1 and uninfected cells (Figure 3B), treatment of DENV-infected cells with heparin lyases and/or chondroitinase ABC did not decrease surface expression of NS1 (Figure 7D). Thus, NS1 expressed on the surface of infected cells is not modulated by GAG expression and must attach by an independent mechanism.

### Soluble DENV NS1 Binds Selectively to Endothelium In Situ

Soluble NS1 has been hypothesized to contribute to DHF pathogenesis by promoting vascular leakage [28,34], which occurs predominantly into pleural and peritoneal cavities [4]. Our cell culture experiments indicated that soluble DENV NS1 binds selectively to subsets of human endothelial cells. We hypothesized, that in vivo, a preferential interaction of DENV NS1 with specific endothelium could contribute to tissue-specific vascular leakage after immune recognition. This mechanism would not require direct DENV infection of endothelial cells, which has been difficult to establish in vivo by pathological criteria [57,58].

**Figure 8:**
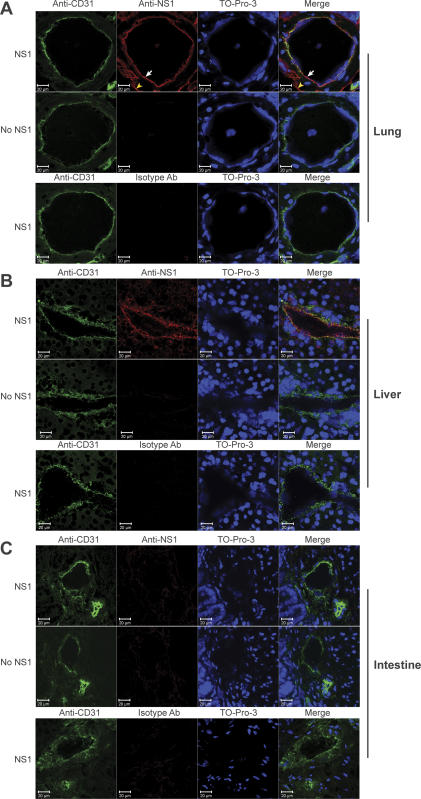
Binding of DENV NS1 to Mouse Tissues Cryo-sections of mouse (A) lung, (B) liver, and (C) intestine were incubated with serum-free supernatants from BHK DENV-2 Rep or BHK cells for 1 h at room temperature. After extensive washing, bound NS1 was detected by a mixture of NS1 mAbs (1A4, 1F11, 2G6, 1B2) followed by Cy3-conjugated goat anti-mouse IgG. Co-staining with endothelial cell marker was subsequently performed by incubating the sections with rat anti-mouse CD31 (PECAM-1) followed by Alexa Fluor 488-conjugated goat anti-rat IgG. Nuclei were stained with a DNA-specific dye TO-PRO-3. Sections incubated with DENV NS1 followed by an isotype control Ab served as a negative control. Analysis was performed by confocal microscopy. White arrow and yellow arrowhead denote the layer of endothelial cells in the lumen and the outer layer of the adventitia of pulmonary vessel, respectively.

**Figure 9:**
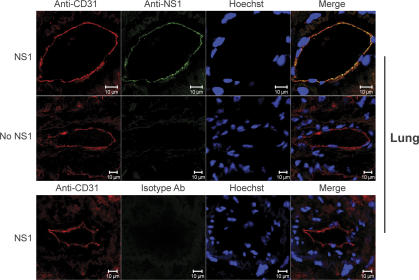
Binding of DENV NS1 to Human Lung Cryo-sections of human lung tissue were incubated with 20 μg/ml of purified DENV-2 NS1 or BSA for 1 h at room temperature. Bound NS1 was detected by staining with a mixture of DENV NS1 mAbs (1A4, 1F11, 2G6, 1B2) followed by Alexa Fluor 488 conjugated with goat anti-mouse IgG. Subsequently, the sections were co-stained with rabbit anti-human CD31 (PECAM-1) followed by Cy3-conjugated donkey anti-rabbit IgG. Nuclei were stained with a DNA-specific dye (Hoechst) and the sections were analyzed by confocal microscopy. Sections incubated with purified DENV NS1 and stained with isotype mAbs served as a negative control.

To assess this, sections of uninfected mouse tissues were incubated with soluble DENV NS1 and analyzed by immunofluorescence and confocal microscopy. Histological analysis was performed on parallel hematoxylin and eosin–stained sections (unpublished data). Specific NS1 binding to endothelial cells lining blood vessels of lung and liver was observed, based on costaining with mAbs against DENV NS1 and the endothelial cell–specific marker CD31 (Figure 8A and 8B). Differences in the pattern of DENV NS1 binding within the same organ were also observed. For example, DENV NS1 bound primarily to endothelial cells lining vessels along the bronchial tree, yet bound poorly to those lining alveolar capillaries. Strong binding of DENV NS1 to the cells lining the outer layer of the adventitia of pulmonary vessels was also apparent (Figure 8A). In the liver, hepatic arteriolar and sinusoidal endothelial cells bound DENV NS1 weakly, whereas those within central veins showed strong binding (Figure 8B). As controls, no appreciable staining was observed if sections were incubated with bovine serum albumin (BSA) followed by anti-NS1 mAbs or with purified DENV NS1 and isotype control mAbs. The selectivity of soluble NS1 binding to endothelium in situ was also demonstrated by an absence of NS1 binding to endothelium in the large intestine (Figure 8C) and brain (unpublished data) of mice. Finally, binding of DENV NS1 to endothelium in situ also was confirmed in human lung tissues (Figure 9).

Next, soluble GAG and DENV NS1 competitive binding assays were performed on lung tissues and images were analyzed by confocal microscopy and Volocity software. Pre-incubation of DENV NS1 with soluble CS-E or HP reduced binding to lung endothelium in situ (CS-E: ∼40%, *p* < 0.0001; HP: ∼30%, *p* = 0.0002). In contrast, CS-C marginally decreased DENV-NS1 attachment to lung endothelium (∼8%, *p* = 0.18). Significant binding of DENV NS1 to serosal surfaces of the lung (pleura) and intestine (peritoneum) was also observed (Figure S2A and S2B). Based on histological analysis, mesothelial cells were also targets for DENV NS1 binding in the lung and intestine. Binding of DENV NS1 to mesothelial cells was specific as negative results were obtained when the sections were incubated with NS1 followed by isotype control mAbs or without NS1 followed by anti-NS1 mAbs. In competitive binding experiments, soluble HP and CS-E, but not CS-C, also decreased DENV NS1 binding to lung mesothelial cells (unpublished data).

## Discussion

In this study, we demonstrate that soluble DENV NS1 binds to a subset of uninfected cells via interactions with GAG, primarily HS and CS-E. In cell culture, NS1 bound strongly to epithelial cells and fibroblasts, and weakly, if at all, to freshly isolated human peripheral blood leukocytes. Substantial variability was observed in NS1 binding to cultured endothelial cells and endothelium in situ. Experiments with the sulfation inhibitor sodium chlorate established that highly sulfated forms of GAG are required for optimal binding of soluble DENV NS1. Finally, our experiments suggest that NS1 on the surface of DENV-infected cells is linked primarily by a distinct, GAG-independent mechanism.

Two major types of GAGs, HS and CS, are produced by cells in the form of proteoglycans as linear polymers of repeating disaccharides of uronic acids and glucosamines or galactosamines. For HS, sulfation of glucosamine and uronic acid moieties may occur in a clustered manner to generate highly sulfated domains or in a dispersed pattern to generate less sulfated or non-sulfated domains. Moreover, GlcNS, IdoA, and GlcA can be sulfated at multiple positions. Differential sulfation of GAG results in preferential binding of growth factors, cytokines, chemokines, enzymes, extracellular matrix, and other proteins to these structures [59]. Cell type–specific expression of GAG modifying enzymes and proteoglycan core proteins results in the display of unique GAG structures on different types of cells [60]. Consistent with this, our studies demonstrate that DENV NS1 binding to GAG occurs in a cell type–specific manner.

The specificity of GAG interactions is determined by the pattern of the disaccharide units, the degree of sulfation, and the spacing of basic amino acid residues in GAG-binding domains of ligands (reviewed in [53–55]). We observed that highly sulfated GAG, including HS, HP, and CS-E, demonstrated the strongest binding for soluble DENV NS1. The use of mutant CHO lines expressing different levels and forms of GAG established that specific HS and CS structures modified by 2-*O* and 3-*O*-sulfotransferases promoted DENV NS1 binding to cell surfaces. These results are analogous, although not identical, to studies with HSV glycoprotein D, which show enhanced cell surface binding when HS is modified by some, but not all 3-OST [50,61]. Sulfation was critical for soluble DENV NS1 binding to cell surfaces, especially for BHK cells, as treatment with the sulfate inhibitor sodium chlorate abolished DENV NS1 attachment.

Also consistent with our results, CS-E, but not CS-A, -B, or -C, interfered with HSV binding to the target cells [62]. Using radioactive binding or gel mobility shift assays, we observed similarly strong binding between West Nile virus NS1 and HS or CS-E (L. Zhang, K. Chung, and M. Diamond, unpublished data). As binding of soluble DENV NS1 to GAG requires sulfation, it is plausible that the interaction is primarily electrostatic in nature and depends on the relative degree of negative charge on a GAG motif. The major repeating disaccharides in HP are IdoA2S-GlcNS6S (∼2.7 sulfates/disaccharides), whereas the major repeating disaccharides in CS-D and CS-E are GlcA2S-GalNAc6S and GlcA-GalNAc4S6S, respectively (∼1.7 sulfates/disaccharide) [62]. Because NS1 preferentially interacts with CS-E and HP compared to CS-D, we hypothesize that NS1 binding may prefer closely spaced sulfates within the same sugar residue. Since HP and CS-E have distinct sugar sequences, the combination of disaccharide unit and position of sulfation on an individual GAG may determine the strength of DENV NS1 binding.

The linear sequences XBBXBX and XBBBXXBX (where B is a basic Arg or Lys amino acid) are common HP binding motifs in proteins [55]. However, GAG binding sites may not be exclusively defined by linear sequences but also can include conformational epitopes that juxtapose basic amino acids from different segments of a protein [55]. Amino acid sequence analysis of DENV NS1 reveals no apparent canonical GAG binding motifs. Although reverse genetic strategies are planned, the identification of the amino acids involved in GAG recognition may await solution of the NS1 structure.

Our data showing that soluble NS1 can bind to the surface of the GAG-deficient line, CHO-745, albeit at significantly lower levels, suggest that additional molecules also serve as ligands for DENV NS1 attachment. This secondary attachment ligand may be expressed only on subsets of cells as addition of sodium chlorate completely abrogated soluble NS1 binding to BHK cells. Consistent with this, soluble HS and CS-E only partially reduced DENV NS1 binding to HMEC. Thus, different cell types may express multiple ligands for attachment of soluble DENV NS1.

The expression of NS1 on the surface of DENV-infected cells was insensitive to treatments that reduce GAG levels. This also suggests the existence of an alternate mechanism of cell surface NS1 attachment. Some have speculated that a transmembrane form of NS1 exists, although the protein sequence lacks a canonical hydrophobic membrane-spanning domain [42]. Others have postulated that at least some fraction of cell surface NS1 is linked via a GPI anchor [43,63]. Based on our data, we hypothesize that DENV NS1 is expressed on cell surfaces by at least two mechanisms: on uninfected cells soluble NS1 binds to the surface via a GAG-dependent and GAG-independent mechanism, whereas on infected cells NS1 attaches via a GAG-independent mechanism, possibly via a membrane or GPI anchor. What remains uncertain, and is a direction for future research, is whether the mechanism of cell surface attachment of NS1 has unique functional consequences, especially in terms of immune recognition or evasion.

Our studies demonstrate that soluble DENV NS1 differentially binds to cultured endothelial cells in vitro and endothelium in situ in human and mouse tissues. In contrast, others have shown that intravenous injection of C57BL/6 x SJL mice with high concentrations (250 μg/ml) of soluble DENV-1 NS1 results in binding to and accumulation in hepatocytes but not to other cell types in the liver or other tissues [40]. A possible reason for the disparity in results is that the route of antigen administration may modulate NS1 binding: intravenous injection could prompt rapid first-pass clearance of antigen by the liver. Although further studies are necessary, our data are consistent with specific GAG modification by subsets of endothelial cells in different tissues modulating the level of bound NS1. Indeed, tissue-specific expression of different isoforms of enzymes in GAG biosynthesis has been reported [64,65]. Selective IL-8 binding to endothelial cells has been observed [66] and could be due to subtle differences in the display of GAG on the surface of cells. In favor of this hypothesis, IL-8 selectively binds to subsets of HP and HS [67].

The pathologic mechanism underlying selective vascular leakage at serosal sites during DHF/DSS [4] remains unknown. High levels of intravascular soluble NS1, as observed in DENV-infected patients, could promote binding and surface expression of NS1 on selective endothelium without a requirement for direct viral infection, which has been difficult to establish histopathologically in fatal DHF cases [57,58]. In addition, specific binding to mesothelial cells that line pleura and peritoneum, as observed in our DENV NS1 binding experiments in situ, could contribute to the pleural effusions or ascites that are observed in DHF/DSS patients. Although more experiments are necessary, preferential binding of soluble NS1 to subsets of endothelial and mesothelial cells in vivo could lead to tissue-specific vascular leakage that occurs during severe secondary DENV infection after recognition by anti-NS1 antibodies, immune complex formation, and inflammatory damage [28,68].

## Materials and Methods

### Reagents.

CS-A, -B, -C, -D, -E, HP, HS, heparin lyases I, II, and III, chondroitinase ABC, anti-heparin lyase-digested HS (3G10), and anti-chondroitinase ABC-digested CS (2B6) antibodies were all purchased (Seikagaku). DENV-2 NS1–specific monoclonal antibodies (mAb hybridomas 2G6, 1A4, 1B2, 1F11, 2E3, and 2E11 [69], and unpublished data) were purified by protein G affinity chromatography. Mouse polyclonal anti-DENV-2 NS1 was produced after BALB/c mice were intraperitoneally immunized three times with purified DENV-2 NS1 (10 μg/dose) at a 2-wk interval. Mice were subsequently treated with pristane followed by injection with myeloma cells to induce ascites formation. Ascites fluid containing anti-NS1 polyclonal antibody was collected, and antibodies were purified by protein-G affinity chromatography. Anti-mouse IgG conjugated with Alexa Fluor 647, Alexa Fluor 488, or Cy3 were purchased from Invitrogen. Sodium chlorate and sodium sulfate were obtained commercially (Sigma).

### Cell culture.

The following transformed cell lines were obtained from the ATCC: BHK fibroblasts cells, African green monkey Vero cells, HEK-293T human embryonic kidney carcinoma cells, L929 mouse fibroblasts, IMR-90 human lung fibroblasts, Detroit-550 human skin fibroblasts, CCD-1102 human keratinocytes, HepG2 and Hep3B human hepatocellular carcinoma cells, 4/4RM4 rat lung mesothelial cells, Eahy926 HUVEC, MEG-01 human megakaryoblast cells, Jurkat human leukemic T lymphoblasts, H9 human T lymphoma cells, EL-4 mouse T lymphoma cells, and U937 human myelomonocyte cells. HMEC and keratinocytes (HaCat) were gifts (M. Caparon). Wild-type CHO-K1 cells and CHO mutant lines with altered GAG expression (CHO-745, CHO-M1, CHO-H8, CHO-K1.5, CHO-K1.N7, CHO-3OST5–1, and CHO-PPP6) have been described previously [46–49,61,70]. BHK, Vero, 293T, L929, and HaCat cells were cultured in Dulbecco's modified Eagle's medium (DMEM) supplemented with 10% fetal bovine serum (FBS), 50 mM HEPES, 4 mM L-glutamine, 100 units/ml penicillin G, and 100 μg/ml streptomycin sulfate. IMR-90, Detroit-551, HepG2, and Hep3B were grown in minimum essential Eagle's medium with Earle's BSS, 10% FBS, 1% non-essential amino acids (NEAA), 1 mM sodium pyruvate, 2 mM L-glutamine, 100 units/ml penicillin G, and 100 μg/ml streptomycin sulfate. CCD-1102 cells were grown in keratinocyte serum-free medium supplemented with keratinocyte growth factors (Invitrogen). MEG-01, U937, Jurkat, and H9 were cultured in RPMI 1640 medium with 10% FBS, 2 mM L-glutamine, 10 mM HEPES, 1 mM sodium pyruvate, 100 units/ml penicillin G, and 100 μg/ml streptomycin sulfate. EL-4 cells were propagated in Iscove's medium supplemented with 10% FBS, 1% non-essential amino acids, 2 mM L-glutamine, 1 mM sodium pyruvate, 100 units/ml penicillin G, and 100 μg/ml streptomycin sulfate. HMEC were grown in MCDB 131 (Invitrogen) supplemented with 10% FBS, 2 mM L-glutamine, 0.3% NaHCO_3_, 1 μg/ml hydrocortisone (Sigma), 10 ng/ml epidermal growth factor (Sigma), 100 units/ml penicillin G, and 100 μg/ml streptomycin sulfate. All CHO cell lines were grown in Ham's F12 medium containing 10% FBS, 100 units/ml penicillin G, and 100 μg/ml streptomycin sulfate, except for CHO-30ST5–1 cells, which were cultured in CHO medium supplemented with 400 μg/ml of G418 sulfate (Cellgro).

Primary human tonsil epithelial cells were isolated according to a published protocol [71] and cultured in keratinocyte serum-free medium supplemented with keratinocyte growth factor (Invitrogen). Primary HMEC-LB and aortic endothelial cells were purchased from Clonetics (Cambrex Bio Science) and maintained according to the manufacturer's protocol. HUVEC were grown in RPMI 1640 containing 10% FBS on 1% gelatin-coated surfaces. Primary human peripheral blood mononuclear cells were isolated from buffy coats obtained from the blood bank or healthy volunteers by Ficoll-Hypaque (Pharmacia) density gradient centrifugation. Human CD14^+^ monocytes, CD19^+^ B cells, CD4^+^ T lymphocytes were purified from primary human peripheral blood mononuclear cells via positive selection using antibody-coated magnetic beads (Miltenyi Biotec). To generate macrophages, monocytes were cultured for 7 d in RPMI 1640 supplemented with 50 ng/ml of GM-CSF, 10% FBS, 2 mM glutamine, 1% nonessential amino acids, 1% sodium pyruvate, 100 units/ml penicillin G, and 100 μg/ml streptomycin sulfate. Human platelets were isolated from whole blood of healthy volunteers. After centrifugation at 750 x *g* for 20 min at 22 °C, platelet-rich plasma was collected and centrifuged at 1,200 x *g* for 15 min at 22 °C to obtain platelets. Human erythrocytes were obtained from healthy donors following a published protocol [72]. All primary cells were cultured for five or fewer passages for the NS1 binding experiments.

### Purification of DENV NS1.

BHK cells that stably propagate a DENV-2 subgenomic replicon (BHK DENV-2 Rep cells [45]) were grown to 80%–90% confluence in DMEM containing 10% FBS and 3 μg/ml puromycin (Sigma). Cell monolayers were washed several times with DMEM and the cells were cultured for another 3 d in serum-free DMEM supplemented with 3 μg/ml puromycin. DENV NS1 secreted in the supernatants of BHK DENV-2 Rep was quantified by NS1 capture ELISA as previously described [28]. Supernatants were collected, centrifuged, pooled, and passed through a 0.2-μm filter prior to immunoaffinity chromatography with anti-NS1 mAb 2G6 [28]. Peak elution fractions were combined, diluted 7-fold with 20 mM Tris (pH 8.0), and loaded onto a 1-ml Mono Q ion exchange column (GE Healthcare) at a rate of 1 ml/min. The protein was eluted with a linear salt gradient (0–1 M NaCl) over 20-column volumes at 1 ml/min. Purity and immunoreactivity of DENV NS1 were confirmed by SDS-PAGE with silver staining and western blot. Concentrations of purified protein were determined by the bicinchoninic acid assay. Purified DENV NS1 from DENV-infected mammalian cells was obtained as previously described [28].

### DENV NS1 binding to cells.

Adherent cells were removed from tissue culture plates after incubation with an EDTA solution (4 mM EDTA plus 10% FBS in PBS). Cells (5 × 10^5^) in suspension were incubated on ice for 1 h with 100 μl of purified DENV NS1 at indicated concentrations or 300 μl of serum-free supernatants from BHK DENV-2 Rep cells. After washing once with 3 ml of medium, 50 μl of DENV-2 NS1 specific mAb 2G6 (25 μg/ml) or an isotype-matched negative control mAb was added to the cells and incubated on ice for 45 min. After subsequent washing, bound primary mAbs were detected after 30-min incubation with a 1:500 dilution of Alexa Fluor 647-conjugated anti-mouse IgG (Invitrogen). Of note, experiments were also performed in parallel with serial washing steps and no significant difference in binding or background was observed. Propidium iodide (0.2 mg/ml) was added immediately before flow cytometry to exclude dead cells.

### Soluble GAG and DENV NS1 competitive binding assays.

Cell suspensions were incubated with DENV NS1 in the presence or absence of varying concentrations of soluble GAG for 1 h on ice. Bound DENV NS1 was detected after antibody staining as described above and analyzed by flow cytometry.

### Enzymatic digestion of GAGs from cellular surfaces.

Cell suspensions were centrifuged at 200 x *g* for 2 min and were resuspended in PBS containing 0.1% BSA. Heparin lyases I, II, III (0.06 U/ml final concentration) or chondroitinase ABC (0.1 U/ml final concentration) was added to a 50-μl of cell suspension and incubated at 37 °C in a shaking incubator for 1 h. After three washes with cold DMEM, the cells were incubated sequentially with DENV NS1 and DENV NS1 mAbs and analyzed by flow cytometry.

### Quantification of surface GAG levels.

Different types of CHO cells were cultured as described above. CHO cells were rinsed and detached from cell culture dishes after incubation in PBS/EDTA for 15 min. Cells (10^7^) were treated with 2 ml trypsin-EDTA (1X solution, Mediatech) at 37 °C for 10 min, which released cell surface proteoglycans. These were collected and used for cell surface GAG isolation and quantification by high-pressure liquid chromatography as reported previously [51].

### DENV NS1 and GAG binding ELISA.

Maxi-Sorp microtiter plates (Nalge Nunc International) were adsorbed with soluble GAG (1 mg/ml in PBS) at 4 °C overnight. After four washes with PBS (300 μl/well), nonspecific binding sites were blocked with 1% heat-inactivated (65 °C, 15 min) BSA in PBS for 2 h at 37 °C and followed by five washes with PBS. Clarified serum-free supernatants from BHK or BHK DENV-2 Rep cells (100 μl diluted 1:1 in PBS) were added to each well and incubated for 2 h at room temperature. Plates were then washed five times with PBS containing 0.05% Tween-20 followed by a 1-h incubation at room temperature with 100 μl of purified DENV NS1 specific mAb mixtures (1A4, 1B2, 1F11, 2G6, and 2E3; 1 μg/ml of each in PBS containing 0.1% BSA). After washing, biotinylated goat anti-mouse IgG (1 μg/ml) and horseradish peroxidase-conjugated streptavidin were added sequentially for 1-h incubations at room temperature. After six final washes with PBS, signal was detected by adding 150 μl of TMB substrate (DakoCytomation) and 50 μl of 0.1 N H_2_SO_4_ stop solution to each well. Plates were evaluated at 450 nm on a 96-well plate reader (Genios Pro; Tecan Instruments).

### Sodium chlorate treatment of cell cultures.

Sulfation was inhibited by sodium chlorate treatment [56]. BHK and CHO cells were cultured in sulfate-free Joklik Modification Minimum Essential Medium Eagle (Sigma) or Ham's F12 medium (Tissue culture support center, Washington University) supplemented with 10% dialyzed FBS (Sigma) containing different concentrations (1–75 mM) of sodium chlorate. In some experiments, 10 mM sodium sulfate was added to replenish sulfate to the cells. After overnight culture, cells were processed for DENV NS1 binding as described above.

### Analysis of NS1 expression on the surface of DENV-infected cells.

BHK cells (1.6 × 10^5^/well) were seeded onto 12-well tissue culture plate (Costar). 24 h later, cells were infected with DENV-2 (strain 16681) at a multiplicity of infection (MOI) of 3. After a 2-h incubation at 37 °C, cell monolayers were extensively washed and cultured in DMEM containing 4% FBS. In some experiments, DENV-infected cells were cultured in medium containing 1, 10, or 100 μg/ml of HP or CS-C or 25 mM sodium chlorate. At specific intervals post-infection, cells were harvested from culture plates with an EDTA solution (4 mM EDTA plus 10% FBS in PBS). DENV NS1 on the surface was determined by incubation of 5 × 10^5^ cells with anti-DENV-2 NS1 mAb clone 2G6 or mouse polyclonal anti-DENV-2 NS1 (20 μg/ml final concentration) for 1 h at 4 °C. After three washes, cells were incubated with Alexa Fluor 647-conjugated anti-mouse IgG (1:500) and analyzed by flow cytometry. In some experiments, DENV-infected cells were treated with a mix of heparin lyases I, II, III or chondroitinase ABC prior to detection of surface NS1.

### DENV NS1 binding to tissues.

Human lung tissue: The protocol to obtain human lung tissues from patients was approved by the Institutional Review Board at the Faculty of Medicine Siriraj Hospital, Mahidol University. A human lung, surgically removed with informed consent from a patient with lung cancer, was evaluated macroscopically for cancer-free regions. Selected tissues, cut from the central part of the cancer-free region of the lung, were cryoprotected in 30% sucrose for generation of 6- to 7-μm frozen sections. Cryosections were thawed, fixed in 4% paraformaldehyde (Sigma) in PBS at room temperature for 10 min, and washed extensively with PBS. Tissues were incubated with purified DENV NS1 or BSA at 20 μg/ml for 1 h at room temperature and followed by three washes with 0.1% BSA in PBS 5 min each. Sections were incubated with a mixture of mAbs (1A4, 1F11, 2G6, 1B2; 20 μg/ml final concentration) against DENV NS1. A mixture of IgG1 (MOPC-21, Sigma) and IgG2a (UPC-10, Sigma) at 20 μg/ml was used as isotype control Abs. After three washes, sections were incubated with Alexa Fluor 488 conjugated goat anti-mouse IgG secondary antibody (Invitrogen) and followed by 1-h incubation at room temperature with rabbit anti-human CD31 (PECAM) (Santa Cruz Biotechnology) at the dilution of 1:10. Sections were washed three times and incubated with Cy3 conjugated donkey anti-rabbit IgG (Jackson Immuno Research Laboratories). Nuclear staining was achieved by incubation for 15 min with 1:100 dilution of Hoechst dye (Invitrogen) and visualized using a Zeiss 510 Meta LSM confocal microscope.

Mouse tissues: To obtain tissues from mice, 4- to 6-wk-old uninfected C57BL/6 mice were anesthesized with ketamine and xylazine and perfused with 20 ml of PBS. Tissues were dissected and cryoprotected in 30% sucrose for generation of frozen sections. Serial 6-μm cryosections were air-dried for 1 h at room temperature followed by washing with PBS. Sections were fixed with 50% acetone in PBS for 10 min on ice, incubated with PBS containing 2 M NaCl (pH 7.4) for 10 min at room temperature, and blocked in PBS containing 0.5% BSA for 1 h at room temperature or overnight at 4 °C. Subsequently, sections were incubated with serum-free supernatants from BHK DENV-2 Rep or BHK cells for 1 h at room temperature followed by incubation with a mixture of anti-NS1-mAbs (1A4, 1F11, 2G6, 1B2; 10 μg/ml final concentration) or isotype control mAbs (10 μg/ml) diluted in PBS containing 0.5% BSA. After three washes, sections were incubated with Cy3 conjugated with goat anti-mouse IgG (Zymed) at the dilution of 1:200. Co-staining with endothelial cell marker, CD31 (PECAM), was accomplished by a 1-h incubation with rat anti-mouse PECAM-1 (CD31) (BD Pharmingen) at a dilution of 1:250 followed by incubation with a 1:500 dilution of secondary Alexa Fluor 488 conjugated anti-rat IgG (Invitrogen). Nuclear staining was achieved by incubation for 15 min with 1:2,500 dilution of TO-Pro-3 (Invitrogen). Sections were mounted using fluorescent mounting medium (Vector Laboratories) and analyzed using a Zeiss 510 Meta LSM confocal microscope. For soluble GAGs and DENV NS1 competitive binding experiments, serum-free supernatants from BHK DENV-2 Rep were mixed with soluble CS-C, CS-E, or HP (3.3 μM final concentration) prior to incubation with sections of mouse lung. The sections were further processed for DENV NS1 and CD31 staining as described above. 50–60 images of each condition were captured by confocal microscopy, and fluorescent intensity of each image was analyzed using the Volocity software (Improvision). Regions that stained positively for CD31 (intensity greater than 427 arbitrary units on the intensity scale of 0–4,095) were selected for analysis. Mean intensity of DENV NS1 binding was calculated for all measured regions. For analysis of DENV NS1 binding to pleura, regions were manually selected based on anatomical sites at the surface of the lung.

### Statistical analysis.

Datasets were compared by a two-tailed, unpaired *t* test. Multiple comparisons were performed using an ANOVA test. Statistical significance was achieved when *p*-values were < 0.05. Data analysis was performed using Prism software (GraphPad).

## Supporting Information

Figure S1 Immunoreactivity of Purified DENV-2 NS1 with mAbs Purified NS1 from BHK DENV-2 Rep cells (rNS1) or DENV-2 infected cells (iNS1) (250 ng/well) was maintained at room temperature or heated (at 95 °C for 5 min) in SDS nonreducing sample buffer immediately prior to 12% SDS-PAGE. Western blot was performed with the individual NS1-specific mAbs 2E11, 2E3, 1F11, 2G6, 1A4, and 1B2. Monomer and dimer forms of NS1 are indicated.
(1.5 MB TIF)

Figure S2 Binding of DENV NS1 to Mesothelial Cells Lining Pleura and Peritoneum Cryo-sections of mouse (A) lung, (B) intestine were incubated with serum-free supernatants from BHK DENV-2 Rep or BHK cells for 1 h at room temperature. After extensive washing, bound NS1 was detected by a mixture of NS1 mAbs (1A4, 1F11, 2G6, 1B2) followed by Cy3-conjugated goat anti-mouse IgG. Costaining with endothelial cell marker was subsequently performed by incubating the sections with rat anti-mouse CD31 (PECAM) followed by Alexa Fluor 488-conjugated goat anti-rat IgG. Nuclei were stained with a DNA-specific dye TO-Pro-3. Sections incubated with DENV NS1 followed by an isotype control Ab served as a negative control. Analysis was performed by confocal microscopy. Arrowheads denote a single layer of mesothelial cells lining pleura (A) or peritoneum (B).
(38.7 MB TIF)

## Abbreviations

2-OST: iduronic/glucuronic acid 2-*O*-sulfotransferase
3-OST: glucosaminyl 3-*O*-sulfotransferase
BHK: baby hamster kidney fibroblast
BHK DENV-2 Rep: BHK DENV-2 subgenomic replicon
BSA: bovine serum albumin
CHO: Chinese hamster ovarian epithelial cell
CS: chondroitin sulfate
DENV: dengue virus
DENV-2: dengue virus serotype 2
DHF/DSS: dengue hemorrhagic fever/dengue shock syndrome
GAG: glycosaminoglycan
GalNAc: N-acetylgalactosamine
GalNAc4S: 4-**O**-sulfated GalNAc
GalNAc4S6S: 4-*O*-and 6-*O*-sulfated GalNAc
GlcA: glucuronic acid
GlcA2S: 2-*O*-sulfated GlcA
GlcNAc: N-acetylglucosamine
GlcNS: N-sulfated glucosamine
GlcNS ± 6S: N-sulfated glucosamine with or without 6-*O*-sulfate
GPI: glycosylphosphatidylinositol
HMEC: human microvascular endothelial cell
HP: heparin
HS: heparan sulfate
HSV: herpes simplex virus
HUVEC: human umbilical cord vein endothelial cell
IdoA: 2-*O*-sulfated IdoA
IdoA: Iduronic acid
MFI: mean fluorescent intensity
NS1: nonstructural protein-1

## References

[ppat-0030183-b001] Gubler DJ (2002). Epidemic dengue/dengue hemorrhagic fever as a public health, social, and economic problem in the 21st century. Trends Microbiol.

[ppat-0030183-b002] Sangkawibha N, Rojanasuphot S, Ahandrik S, Viriyapongse S, Jatanasen S (1984). Risk factors in dengue shock syndrome: a prospective epidemiologic study in Rayong, Thailand. I. The 1980 outbreak. Am J Epidemiol.

[ppat-0030183-b003] Guzman MG, Kouri G, Valdes L, Bravo J, Alvarez M (2000). Epidemiologic studies on dengue in Santiago de Cuba, 1997. Am J Epidemiol.

[ppat-0030183-b004] Nimmannitya S (1987). Clinical spectrum and management of dengue haemorrhagic fever. Southeast Asian J Trop Med Public Health.

[ppat-0030183-b005] Rothman AL, Ennis FA (1999). Immunopathogenesis of dengue hemorrhagic fever. Virology.

[ppat-0030183-b006] Rothman AL (2004). Dengue: defining protective versus pathologic immunity. J Clin Invest.

[ppat-0030183-b007] Halstead SB (1988). Pathogenesis of dengue: challenges to molecular biology. Science.

[ppat-0030183-b008] Halstead SB, O'Rourke EJ (1977). Dengue viruses and mononuclear phagocytes. I. Infection enhancement by non-neutralizing antibody. J Exp Med.

[ppat-0030183-b009] Kliks SC, Nimmanitya S, Nisalak A, Burke DS (1988). Evidence that maternal dengue antibodies are important in the development of dengue hemorrhagic fever in infants. Am J Trop Med Hyg.

[ppat-0030183-b010] Kliks SC, Nisalak A, Brandt WE, Wahl L, Burke DS (1989). Antibody-dependent enhancement of dengue virus growth in human monocytes as a risk factor for dengue hemorrhagic fever. Am J Trop Med Hyg.

[ppat-0030183-b011] Libraty DH, Young PR, Pickering D, Endy TP, Kalayanarooj S (2002). High circulating levels of the dengue virus nonstructural protein NS1 early in dengue illness correlate with the development of dengue hemorrhagic fever. J Infect Dis.

[ppat-0030183-b012] Murgue B, Roche C, Chungue E, Deparis X (2000). Prospective study of the duration and magnitude of viraemia in children hospitalized during the 1996–1997 dengue-2 outbreak in French Polynesia. J Med Virol.

[ppat-0030183-b013] Vaughn DW, Green S, Kalayanarooj S, Innis BL, Nimmannitya S (2000). Dengue viremia titer, antibody response pattern, and virus serotype correlate with disease severity. J Infect Dis.

[ppat-0030183-b014] Leitmeyer KC, Vaughn DW, Watts DM, Salas R, Villalobos I (1999). Dengue virus structural differences that correlate with pathogenesis. J Virol.

[ppat-0030183-b015] Cologna R, Rico-Hesse R (2003). American genotype structures decrease dengue virus output from human monocytes and dendritic cells. J Virol.

[ppat-0030183-b016] Pryor MJ, Carr JM, Hocking H, Davidson AD, Li P (2001). Replication of dengue virus type 2 in human monocyte-derived macrophages: comparisons of isolates and recombinant viruses with substitutions at amino acid 390 in the envelope glycoprotein. Am J Trop Med Hyg.

[ppat-0030183-b017] Watts DM, Porter KR, Putvatana P, Vasquez B, Calampa C (1999). Failure of secondary infection with American genotype dengue 2 to cause dengue haemorrhagic fever. Lancet.

[ppat-0030183-b018] Messer WB, Vitarana UT, Sivananthan K, Elvtigala J, Preethimala LD (2002). Epidemiology of dengue in Sri Lanka before and after the emergence of epidemic dengue hemorrhagic fever. Am J Trop Med Hyg.

[ppat-0030183-b019] Green S, Vaughn DW, Kalayanarooj S, Nimmannitya S, Suntayakorn S (1999). Elevated plasma interleukin-10 levels in acute dengue correlate with disease severity. J Med Virol.

[ppat-0030183-b020] Green S, Vaughn DW, Kalayanarooj S, Nimmannitya S, Suntayakorn S (1999). Early immune activation in acute dengue illness is related to development of plasma leakage and disease severity. J Infect Dis.

[ppat-0030183-b021] Hober D, Poli L, Roblin B, Gestas P, Chungue E (1993). Serum levels of tumor necrosis factor-alpha (TNF-alpha), interleukin-6 (IL-6), and interleukin-1 beta (IL-1 beta) in dengue-infected patients. Am J Trop Med Hyg.

[ppat-0030183-b022] Hober D, Delannoy AS, Benyoucef S, De Groote D, Wattre P (1996). High levels of sTNFR p75 and TNF alpha in dengue-infected patients. Microbiol Immunol.

[ppat-0030183-b023] Hober D, Nguyen TL, Shen L, Ha DQ, Huong VT (1998). Tumor necrosis factor alpha levels in plasma and whole-blood culture in dengue-infected patients: relationship between virus detection and pre-existing specific antibodies. J Med Virol.

[ppat-0030183-b024] Bethell DB, Flobbe K, Cao XT, Day NP, Pham TP (1998). Pathophysiologic and prognostic role of cytokines in dengue hemorrhagic fever. J Infect Dis.

[ppat-0030183-b025] Mongkolsapaya J, Dejnirattisai W, Xu XN, Vasanawathana S, Tangthawornchaikul N (2003). Original antigenic sin and apoptosis in the pathogenesis of dengue hemorrhagic fever. Nat Med.

[ppat-0030183-b026] Mongkolsapaya J, Duangchinda T, Dejnirattisai W, Vasanawathana S, Avirutnan P (2006). T cell responses in dengue hemorrhagic fever: are cross-reactive T cells suboptimal?. J Immunol.

[ppat-0030183-b027] Bashyam HS, Green S, Rothman AL (2006). Dengue virus-reactive CD8+ T cells display quantitative and qualitative differences in their response to variant epitopes of heterologous viral serotypes. J Immunol.

[ppat-0030183-b028] Avirutnan P, Punyadee N, Noisakran S, Komoltri C, Thiemmeca S (2006). Vascular leakage in severe dengue virus infections: a potential role for the nonstructural viral protein NS1 and complement. J Infect Dis.

[ppat-0030183-b029] Lindenbach BD, Rice CM (1999). Genetic interaction of flavivirus nonstructural proteins NS1 and NS4A as a determinant of replicase function. J Virol.

[ppat-0030183-b030] Mackenzie JM, Jones MK, Young PR (1996). Immunolocalization of the dengue virus nonstructural glycoprotein NS1 suggests a role in viral RNA replication. Virology.

[ppat-0030183-b031] Winkler G, Maxwell SE, Ruemmler C, Stollar V (1989). Newly synthesized dengue-2 virus nonstructural protein NS1 is a soluble protein but becomes partially hydrophobic and membrane-associated after dimerization. Virology.

[ppat-0030183-b032] Flamand M, Megret F, Mathieu M, Lepault J, Rey FA (1999). Dengue virus type 1 nonstructural glycoprotein NS1 is secreted from mammalian cells as a soluble hexamer in a glycosylation-dependent fashion. J Virol.

[ppat-0030183-b033] Alcon S, Talarmin A, Debruyne M, Falconar A, Deubel V (2002). Enzyme-linked immunosorbent assay specific to dengue virus type 1 nonstructural protein NS1 reveals circulation of the antigen in the blood during the acute phase of disease in patients experiencing primary or secondary infections. J Clin Microbiol.

[ppat-0030183-b034] Young PR, Hilditch PA, Bletchly C, Halloran W (2000). An antigen capture enzyme-linked immunosorbent assay reveals high levels of the dengue virus protein NS1 in the sera of infected patients. J Clin Microbiol.

[ppat-0030183-b035] Falconar AK (1997). The dengue virus nonstructural-1 protein (NS1) generates antibodies to common epitopes on human blood clotting, integrin/adhesin proteins and binds to human endothelial cells: potential implications in haemorrhagic fever pathogenesis. Arch Virol.

[ppat-0030183-b036] Chang HH, Shyu HF, Wang YM, Sun DS, Shyu RH (2002). Facilitation of cell adhesion by immobilized dengue viral nonstructural protein 1 (NS1): arginine-glycine-aspartic acid structural mimicry within the dengue viral NS1 antigen. J Infect Dis.

[ppat-0030183-b037] Lin CF, Lei HY, Shiau AL, Liu HS, Yeh TM (2002). Endothelial cell apoptosis induced by antibodies against dengue virus nonstructural protein 1 via production of nitric oxide. J Immunol.

[ppat-0030183-b038] Lin CF, Lei HY, Shiau AL, Liu CC, Liu HS (2003). Antibodies from dengue patient sera cross-react with endothelial cells and induce damage. J Med Virol.

[ppat-0030183-b039] Lin CF, Chiu SC, Hsiao YL, Wan SW, Lei HY (2005). Expression of cytokine, chemokine, and adhesion molecules during endothelial cell activation induced by antibodies against dengue virus nonstructural protein 1. J Immunol.

[ppat-0030183-b040] Alcon-LePoder S, Drouet MT, Roux P, Frenkiel MP, Arborio M (2005). The secreted form of dengue virus nonstructural protein NS1 is endocytosed by hepatocytes and accumulates in late endosomes: implications for viral infectivity. J Virol.

[ppat-0030183-b041] Chung KM, Liszewski MK, Nybakken G, Davis AE, Townsend RR (2006). West Nile virus nonstructural protein NS1 inhibits complement activation by binding the regulatory protein factor H. Proc Natl Acad Sci U S A.

[ppat-0030183-b042] Wright PJ, Cauchi MR, Ng ML (1989). Definition of the carboxy termini of the three glycoproteins specified by dengue virus type 2. Virology.

[ppat-0030183-b043] Jacobs MG, Robinson PJ, Bletchly C, Mackenzie JM, Young PR (2000). Dengue virus nonstructural protein 1 is expressed in a glycosyl-phosphatidylinositol-linked form that is capable of signal transduction. FASEB J.

[ppat-0030183-b044] Schlesinger JJ, Brandriss MW, Putnak JR, Walsh EE (1990). Cell surface expression of yellow fever virus nonstructural glycoprotein NS1: consequences of interaction with antibody. J Gen Virol.

[ppat-0030183-b045] Whitby K, Pierson TC, Geiss B, Lane K, Engle M (2005). Castanospermine, a potent inhibitor of dengue virus infection in vitro and in vivo. J Virol.

[ppat-0030183-b046] Esko JD, Stewart TE, Taylor WH (1985). Animal cell mutants defective in glycosaminoglycan biosynthesis. Proc Natl Acad Sci U S A.

[ppat-0030183-b047] Broekelmann TJ, Kozel BA, Ishibashi H, Werneck CC, Keeley FW (2005). Tropoelastin interacts with cell-surface glycosaminoglycans via its COOH-terminal domain. J Biol Chem.

[ppat-0030183-b048] Zhang L, Lawrence R, Frazier BA, Esko JD (2006). CHO glycosylation mutants: proteoglycans. Methods Enzymol.

[ppat-0030183-b049] Zhang L, Beeler DL, Lawrence R, Lech M, Liu J (2001). 6-O-sulfotransferase-1 represents a critical enzyme in the anticoagulant heparan sulfate biosynthetic pathway. J Biol Chem.

[ppat-0030183-b050] Shukla D, Liu J, Blaiklock P, Shworak NW, Bai X (1999). A novel role for 3-O-sulfated heparan sulfate in herpes simplex virus 1 entry. Cell.

[ppat-0030183-b051] Studelska DR, Giljum K, McDowell LM, Zhang L (2006). Quantification of glycosaminoglycans by reversed-phase HPLC separation of fluorescent isoindole derivatives. Glycobiology.

[ppat-0030183-b052] Kinoshita A, Yamada S, Haslam SM, Morris HR, Dell A (1997). Novel tetrasaccharides isolated from squid cartilage chondroitin sulfate E contain unusual sulfated disaccharide units GlcA(3-O-sulfate)beta1-3GalNAc(6-O-sulfate) or GlcA(3-O-sulfate)beta1-3GalNAc. J Biol Chem.

[ppat-0030183-b053] Turnbull J, Powell A, Guimond S (2001). Heparan sulfate: decoding a dynamic multifunctional cell regulator. Trends Cell Biol.

[ppat-0030183-b054] Rosenberg RD, Shworak NW, Liu J, Schwartz JJ, Zhang L (1997). Heparan sulfate proteoglycans of the cardiovascular system. Specific structures emerge but how is synthesis regulated?. J Clin Invest.

[ppat-0030183-b055] Hileman RE, Fromm JR, Weiler JM, Linhardt RJ (1998). Glycosaminoglycan-protein interactions: definition of consensus sites in glycosaminoglycan binding proteins. Bioessays.

[ppat-0030183-b056] Baeuerle PA, Huttner WB (1986). Chlorate: a potent inhibitor of protein sulfation in intact cells. Biochem Biophys Res Commun.

[ppat-0030183-b057] Bhamarapravati N, Tuchinda P, Boonyapaknavik V (1967). Pathology of Thailand haemorrhagic fever: a study of 100 autopsy cases. Ann Trop Med Parasitol.

[ppat-0030183-b058] Jessie K, Fong MY, Devi S, Lam SK, Wong KT (2004). Localization of dengue virus in naturally infected human tissues by immunohistochemistry and in situ hybridization. J Infect Dis.

[ppat-0030183-b059] Esko JD, Selleck SB (2002). Order out of chaos: assembly of ligand binding sites in heparan sulfate. Annu Rev Biochem.

[ppat-0030183-b060] Bishop JR, Schuksz M, Esko JD (2007). Heparan sulphate proteoglycans fine-tune mammalian physiology. Nature.

[ppat-0030183-b061] Xia G, Chen J, Tiwari V, Ju W, Li JP (2002). Heparan sulfate 3-O-sulfotransferase isoform 5 generates both an antithrombin-binding site and an entry receptor for herpes simplex virus, type 1. J Biol Chem.

[ppat-0030183-b062] Bergefall K, Trybala E, Johansson M, Uyama T, Naito S (2005). Chondroitin sulfate characterized by the E-disaccharide unit is a potent inhibitor of herpes simplex virus infectivity and provides the virus binding sites on gro2C cells. J Biol Chem.

[ppat-0030183-b063] Noisakran S, Dechtawewat T, Rinkaewkan P, Puttikhunt C, Kanjanahaluethai A (2007). Characterization of dengue virus NS1 stably expressed in 293T cell lines. J Virol Methods.

[ppat-0030183-b064] Turnbull J, Drummond K, Huang Z, Kinnunen T, Ford-Perriss M (2003). Heparan sulphate sulphotransferase expression in mice and Caenorhabditis elegans. Biochem Soc Trans.

[ppat-0030183-b065] Zhang L, Schwartz JJ, Miller J, Liu J, Fritze LM (1998). The retinoic acid and cAMP-dependent up-regulation of 3-O-sulfotransferase-1 leads to a dramatic augmentation of anticoagulantly active heparan sulfate biosynthesis in F9 embryonal carcinoma cells. J Biol Chem.

[ppat-0030183-b066] Rot A, Hub E, Middleton J, Pons F, Rabeck C (1996). Some aspects of IL-8 pathophysiology. III: Chemokine interaction with endothelial cells. J Leukoc Biol.

[ppat-0030183-b067] Witt DP, Lander AD (1994). Differential binding of chemokines to glycosaminoglycan subpopulations. Curr Biol.

[ppat-0030183-b068] Avirutnan P, Malasit P, Seliger B, Bhakdi S, Husmann M (1998). Dengue virus infection of human endothelial cells leads to chemokine production, complement activation, and apoptosis. J Immunol.

[ppat-0030183-b069] Puttikhunt C, Kasinrerk W, Srisa-ad S, Duangchinda T, Silakate W (2003). Production of anti-dengue NS1 monoclonal antibodies by DNA immunization. J Virol Methods.

[ppat-0030183-b070] Duncan MB, Chen J, Krise JP, Liu J (2004). The biosynthesis of anticoagulant heparan sulfate by the heparan sulfate 3-O-sulfotransferase isoform 5. Biochim Biophys Acta.

[ppat-0030183-b071] Pegtel DM, Middeldorp J, Thorley-Lawson DA (2004). Epstein-Barr virus infection in ex vivo tonsil epithelial cell cultures of asymptomatic carriers. J Virol.

[ppat-0030183-b072] Pattanapanyasat K, Noulsri E, Fucharoen S, Lerdwana S, Lamchiagdhase P (2004). Flow cytometric quantitation of red blood cell vesicles in thalassemia. Cytometry B Clin Cytom.

[ppat-0030183-b073] Diamond MS, Edgil D, Roberts TG, Lu B, Harris E (2000). Infection of human cells by dengue virus is modulated by different cell types and viral strains. J Virol.

[ppat-0030183-b074] Lawrence R, Kuberan B, Lech M, Beeler DL, Rosenberg RD (2004). Mapping critical biological motifs and biosynthetic pathways of heparan sulfate. Glycobiology.

[ppat-0030183-b075] Liu J, Shriver Z, Pope RM, Thorp SC, Duncan MB (2002). Characterization of a heparan sulfate octasaccharide that binds to herpes simplex virus type 1 glycoprotein D. J Biol Chem.

